# Histone methyltransferase Smyd2 contributes to blood‐brain barrier breakdown in stroke

**DOI:** 10.1002/ctm2.761

**Published:** 2022-03-16

**Authors:** Jinghuan Wang, Wen Zhong, Qianwen Cheng, Chenxi Xiao, Jie Xu, Zhenghua Su, Haibi Su, Xinhua Liu

**Affiliations:** ^1^ Pharmacophenomics Laboratory Human Phenome Institute Pharmacy School Fudan University Shanghai China

**Keywords:** blood‐brain barrier, brain microvascular endothelial cells, ischaemia stroke, Smyd2

## Abstract

**Background:**

The blood‐brain barrier (BBB) plays a principal role in the healthy and diseased central nervous systems, and BBB disruption after ischaemic stroke is responsible for increased mortality. Smyd2, a member of the SMYD‐methyltransferase family, plays a vital role in disease by methylation of diverse substrates; however, little is known about its role in the pathophysiology of the brain in response to ischaemia‐reperfusion injury.

**Methods:**

Using oxygen glucose deprivation and reoxygenation (OGD/R)‐induced primary brain microvascular endothelial cells (BMECs) and Smyd2 knockdown mice subjected to middle cerebral artery occlusion, we evaluated the role of Smyd2 in BBB disruption. We performed loss‐of‐function and gain‐of‐function studies to investigate the biological function of Smyd2 in ischaemic stroke.

**Results:**

We found that Smyd2 was a critical factor for regulating brain endothelial barrier integrity in ischaemia‐reperfusion injury. Smyd2 is upregulated in peri‐ischaemic brains, leading to BBB disruption via methylation‐mediated Sphk/S1PR. Knockdown of Smyd2 in mice reduces BBB permeability and improves functional recovery. Using OGD/R‐induced BMECs, we demonstrated that Sphk/S1PR methylation modification by Smyd2 affects ubiquitin‐dependent degradation and protein stability, which may disrupt endothelial integrity. Moreover, overexpression of Smyd2 can damage endothelial integrity through Sphk/S1PR signalling.

**Conclusions:**

Overall, these results reveal a novel role for Smyd2 in BBB disruption in ischaemic stroke, suggesting that Smyd2 may represent a new therapeutic target for ischaemic stroke.

## Graphical abstract

- Smyd2 is upregulated in peri‐ischaemic brains after ischaemia‐reperfusion injury.
- Increased Smyd2 levels disrupts BBB integrity via Sphk/S1PR pathway and results in neuronal dysfunction.
- Knockdown of Smyd2 in mice reduces BBB permeability and improves functional recovery.

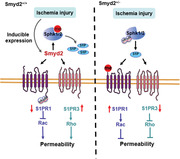

## 1 BACKGROUND

Stroke is a complex and devastating neurological condition that is the third leading cause of death worldwide and the most common cause of disability.^1^ The blood‐brain barrier (BBB) restricts the transport of substances between the circulating blood and the central nervous system (CNS) and plays an essential role in brain homeostasis.^2^, ^3^ BBB dysfunction is recognised as the most devastating complication after an ischaemic stroke and may lead to vasogenic oedema,^4^ which causes high morbidity and mortality in cerebral ischaemia reperfusion (I/R).^5^ Therefore, the elucidation of the mechanisms that regulate BBB integrity and assessment of the potential protection of the BBB are indispensable for the management of CNS diseases.

The BBB consists of brain microvascular endothelial cells (BMECs) with tight junctions (TJs), pericytes, astrocytes and perivascular microglia.^3^, ^6^ During ischaemic stroke progression, interruption or severe reduction in blood supply causes a reduction in TJs protein levels and BBB impairments,^7^, ^8^ which can eventually lead to neurological deficits and poor clinical prognosis.^5^, ^9^ Thus, it is worth investigating how endothelial cells respond to ischaemic injury for developing therapies that may halt disease progression.

Histone methyltransferases (HMTs) are important epigenetic regulators that catalyse the methylation of histone proteins.^10^ SET and MYND domain‐containing protein 2 (Smyd2) is a member of the SMYD‐methyltransferase family that catalyses the methylation of lysine 36 and 4 (H3K36 and H3K4) sites on histones.^11^ Most studies have demonstrated that Smyd2 can also catalyse the methylation of lysine sites on non‐histone proteins, to affect cell proliferation, differentiation and survival.^12^, ^13^, ^14^, ^15^ Thus, diverse methylated substrates mediate the function of Smyd2 in diseases, such as cardiovascular disease and cancer; however, little is known about its function in other diseases. Thus, further investigation into how Smyd2 regulates brain pathophysiology in response to I/R injury is needed.

Bioactive sphingolipid sphingosine‐1‐phosphate (S1P), produced by sphingosine kinases (Sphk1 and Sphk2),^16^ exerts many biological functions through its receptor, S1PR1‐5.^17^ Although all S1PRs are G protein‐coupled, each receptor subtype exhibits diverse cellular responses, through the activation of different downstream signalling pathways. In endothelial cells, S1PR1‐3‐mediated S1P signalling potently regulates endothelial permeability and activation, which are critical events during inflammation and endothelial response to injury.^18^, ^19^ S1PR1‐3 differentially regulate endothelial function. For instance, S1PR1 couples only with Gi/o to activate the GTPase Rac pathway, which plays a critical role in adherens junction and cortical actin assembly, promoting endothelial barrier integrity.^20^, ^21^ In sharp contrast, specific stimuli may favour S1P signalling via S1PR3 or S1PR2 and subsequent activation of the Gq/11 and G12/13 pathways, which can induce endothelial permeability and inflammation.^18^, ^22^ Thus, S1PRs elicit multiple endothelial responses due to the coupling of these receptors to distinct and opposing signalling pathways.

In the present study, we addressed the important role of endothelial cell Smyd2 in Sphk/S1PR signalling during cerebral ischaemia and showed that Smyd2 controls endothelial permeability by regulating Sphk/ S1PR methylation in ischaemic stroke. To assess the importance of Smyd2 in ischaemic injury in vivo, we generated Smyd2 knockdown mice and found that they were resistant to ischaemic injury. Our results suggest new avenues for the development of stroke therapeutics, through elucidating the processes of Smyd2‐mediated BBB disruption in perfusion injury.

## 2 RESULTS

### 2.1 Smyd2 knockdown reduces BBB breakdown and improves stroke outcomes following experimental ischaemic stroke

**FIGURE 1:**
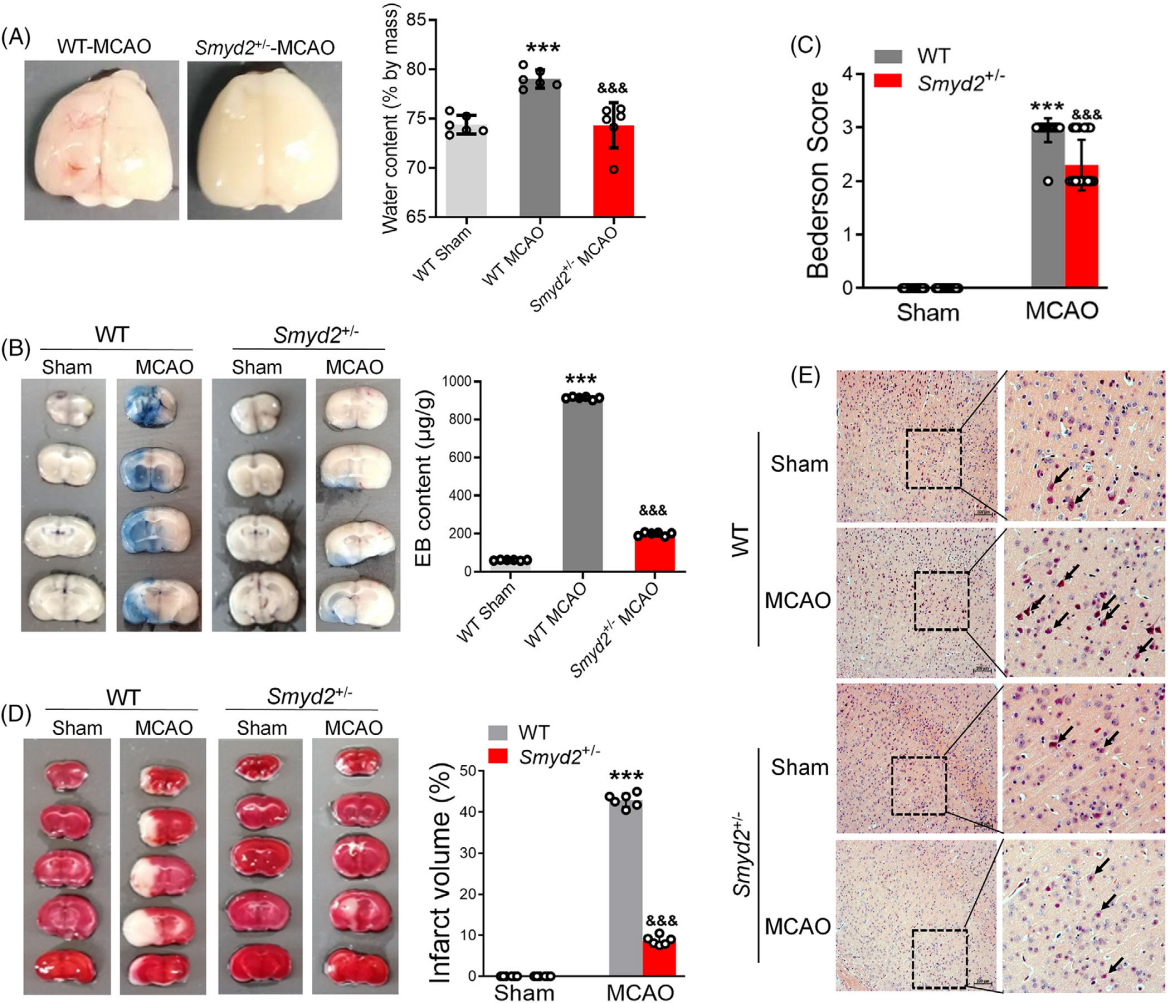
Smyd2 knockdown reduces BBB breakdown and improves stroke outcomes following experimental ischemic stroke. WT and *Smyd2* ^+/–^ mice were subjected to 90 min of MCAO and 24 h of reperfusion. Representative images of brain oedema and water content (*n* = 6) (A); Evans blue‐stained brain sections after MCAO, with Evans blue content expressed as μg/g of brain tissue (*n* = 6) (B); the neurological deficit scores of mice after MCAO (*n* = 10) (C); TTC staining was performed to identify the ischemic regions (*n* = 6) (D); representative images of H&E staining, with representative magnified images shown. Scale bars: 100 μm (E). All data are presented as mean ± SD; ^***^ *p* < .001, compared with the WT‐Sham group, ^&&&^ *p* < .001, compared with the WT‐MCAO group

To understand the role of Smyd2 in BBB breakdown during brain ischaemia and reperfusion, we subjected WT and *Smyd2* ^+/–^ mice to 90 min of transient middle cerebral artery occlusion (MCAO) and 24 h of reperfusion. Apparent cerebral oedemas were observed in the brain samples from WT mice, whereas Smyd2 knockdown noticeably reduced brain swelling (Figure 1A). The BBB permeability was observed using quantitative Evans blue (EB). Representative EB staining images and EB content demonstrated that Smyd2 knockdown decreased BBB permeability (Figure 1B). Accordingly, *Smyd2* ^+/–^ mice showed a decreased neurological deficit score (Figure 1C) and infarct size (Figure 1D) compared to WT mice. To confirm further pathological changes in the stroke brains, haematoxylin and eosin staining was performed. We observed a large number of pyknotic nuclei in selected cortical areas of WT mice post‐stroke. Furthermore, more morphologically intact neurons were observed in the brain areas of *Smyd2* ^+/–^ mice (Figure 1E). These data clearly demonstrate that Smyd2 knockdown reduces BBB breakdown and improves outcomes after ischaemic stroke.

### 2.2 Smyd2 knockdown reduces apoptosis and inflammatory responses following experimental ischaemic stroke

**FIGURE 2:**
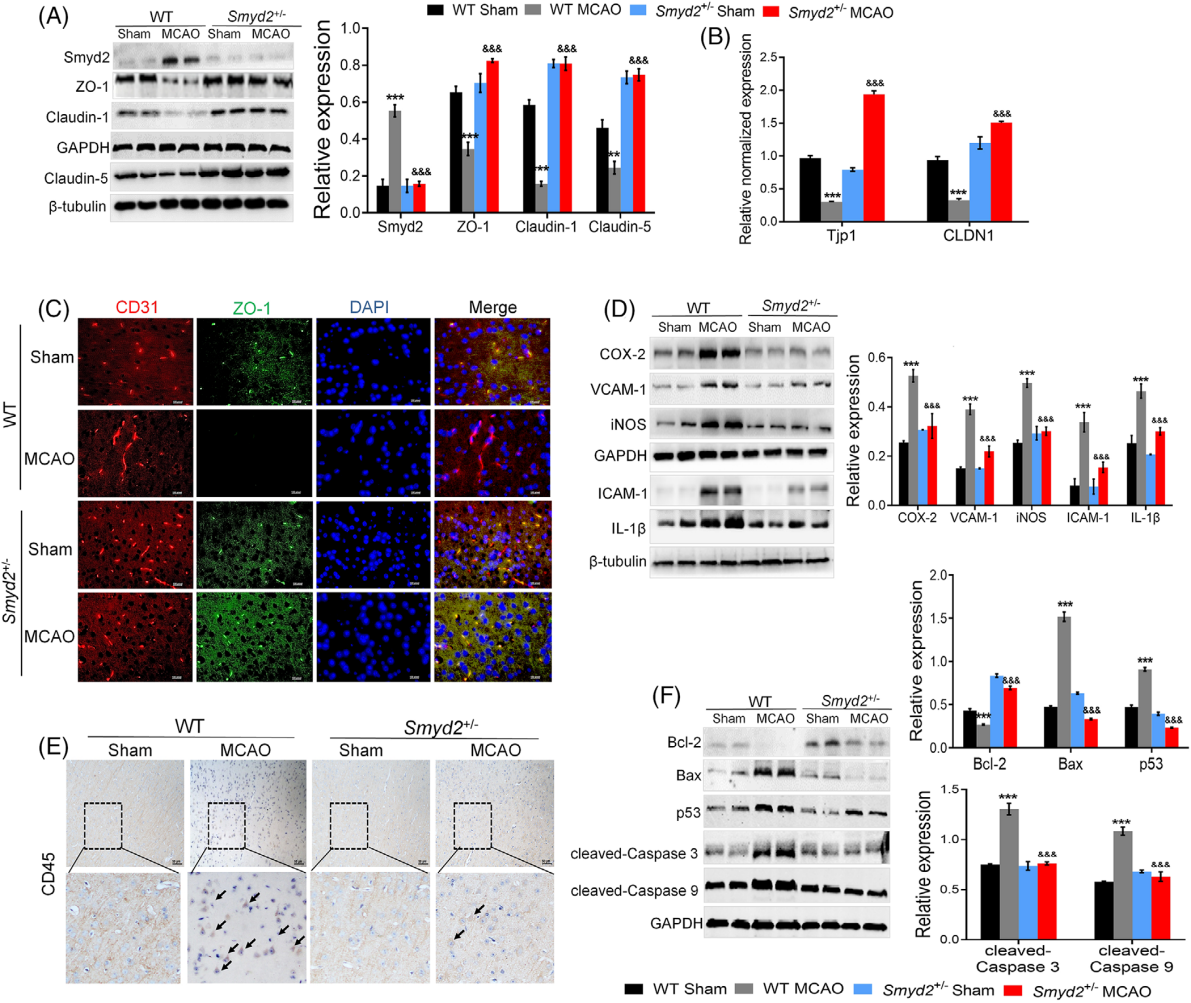
Smyd2 knockdown reduces apoptosis and the inflammatory response. WT and *Smyd2* ^+/–^ mice were subjected to 90 min of MCAO and 24 h of reperfusion. Immunoblot analysis of Smyd2 and TJs‐associated proteins (claudin‐1, ZO‐1, claudin‐5) in the brain of mice (A); qRT‐PCR analysis of TJs‐associated genes (*Tjp1*, *CLDN1*) in the brain of mice (B); immunofluorescence staining of CD31 and ZO‐1 in the brain of mice after MCAO, magnification ×200 (C); immunoblot analysis of inflammation markers (iNOS, VCAM‐1, COX‐2, ICAM‐1, IL‐1β) in the brain of mice after MCAO (D); immunohistochemistry staining of CD45 in the brain of mice. Scale bars: 50 μm (E); immunoblot analysis of apoptosis‐related proteins (Bax, Bcl‐2, p53, cleaved‐Caspase 3, cleaved‐Caspase 9) in the brain of mice (F). All data are presented as mean ± SD, *n* = 6; ^**^ *p* < .01, ^***^ *p* < .001, compared with the WT‐Sham group, ^&&&^ *p* < .001, compared with the WT‐MCAO group

Endothelial TJs are characterised by the presence of proteins, such as ZO‐1 and Claudins, and form a physical barrier that limits the diffusion of blood‐borne molecules into the brain's parenchyma.^23^ The loss of Smyd2 protein in the brains of *Smyd2* ^+/–^ mice was confirmed by western blotting (Figure 2A). We explored the effects of Smyd2 knockdown on the expression of Claudin‐1/5 and ZO‐1 after MCAO and found that Smyd2 knockdown reversed the decrease in Claudin‐1/5 and ZO‐1 after ischaemic stroke (Figure 2A and B). Immunofluorescence staining also revealed that Smyd2 knockdown restored the expression of ZO‐1 and Claudin‐1 (Figures 2C and S1A). There is evidence that inflammatory processes are involved in brain damage after ischaemic stroke, and we found that the MCAO‐induced upregulation of inflammatory mediators were inhibited by Smyd2 knockdown (Figures 2D and S1B). After ischaemia‐reperfusion injury, leukocyte infiltration inside the ischaemic tissue obviously increased, but Smyd2 knockdown decreased leukocyte infiltration in the post‐ischaemic brain by labelling CD45 positive cells (Figure 2E). Since neural cell apoptosis generally contributes to neuropathogenesis after reperfusion, we further investigated the role of Smyd2 knockdown in regulating neural cell apoptosis. The results showed that Smyd2 knockdown reversed the upregulation of pro‐apoptotic proteins, such as Bax and p53, in the brain tissues of I/R mice, whereas it promoted the expression of the anti‐apoptotic factor Bcl‐2 (Figure 2F). RT‐qPCR results confirmed these findings (Figure S1C). Taken together, these data reveal that Smyd2 knockdown offers profound protection in ischaemic brains.

### 2.3 Inhibition of Smyd2 reduces endothelial cell inflammation and barrier disruption after OGD/R

**FIGURE 3:**
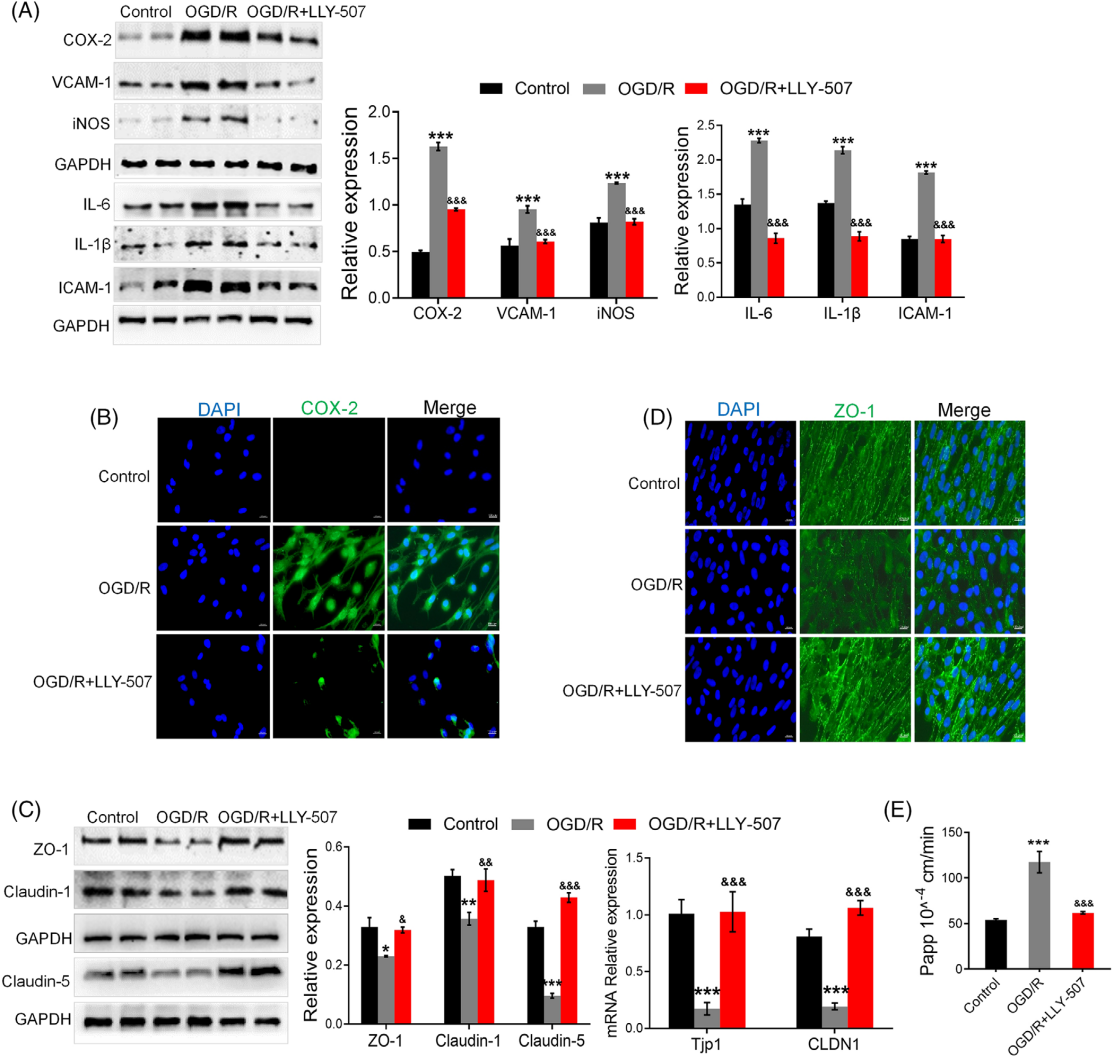
Inhibition of Smyd2 reduces the inflammatory responses and endothelial cell barrier disruption after OGD/R. BMECs were pre‐treated with 2 μmol/L LLY‐507 for 4 h and subsequently subjected to oxygen‐glucose deprivation for 4 h followed by 24 h of reoxygenation (OGD/R). Immunoblot analysis of inflammation markers (iNOS, VCAM‐1, COX‐2, IL‐6, IL‐1β, ICAM‐1) (A); immunofluorescence staining of COX‐2, magnification ×200 (B); immunoblot analysis and RT‐qPCR of TJs‐associated proteins and mRNA expression (C); immunofluorescence staining of ZO‐1, magnification ×200 (D); Na‐F permeability was detected (E). All data are presented as mean ± SD of four independent experiments; ^*^ *p* < .05, ^**^ *p* < .01, ^***^ *p* < .001, compared with the control group, ^&^ *p* < .05, ^&&^ *p* < .01, ^&&&^ *p* < .001 compared with OGD/R group

To address the effects of Smyd2 on I/R‐induced BBB breakdown, we used an in vitro BBB model. Monolayer of rat BMECs was subjected to oxygen‐glucose deprivation and reoxygenation (OGD/R). Figure 3A showed that Smyd2 inhibition by LLY‐507 reversed the upregulation of inflammatory markers (Figure 3A). Immunofluorescence analyses revealed that the inhibition of Smyd2 reduced the expression of COX‐2 (Figure 3B). Because tight junction proteins play an essential role in maintaining the integrity of the endothelial cell barrier, we further determined the expression levels of Claudin‐1/5 and ZO‐1. The results showed that LLY‐507 recovered the decreased levels of ZO‐1 and Claudin‐1/5 (Figure 3C). Consistent with these findings, immunofluorescence staining revealed that LLY‐507 enhanced the expression of ZO‐1 (Figure 3D). Accordingly, Na‐F permeability analysis revealed that inhibition of Smyd2 with LLY‐507 markedly reduced barrier leakage after OGD/R (Figure 3E). Based on these results, the in vitro data confirm the function of Smyd2 in endothelial cell barrier disruption.

### 2.4 Loss of endothelial Smyd2 suppresses inflammatory responses and endothelial cells barrier disruption after OGD/R

**FIGURE 4:**
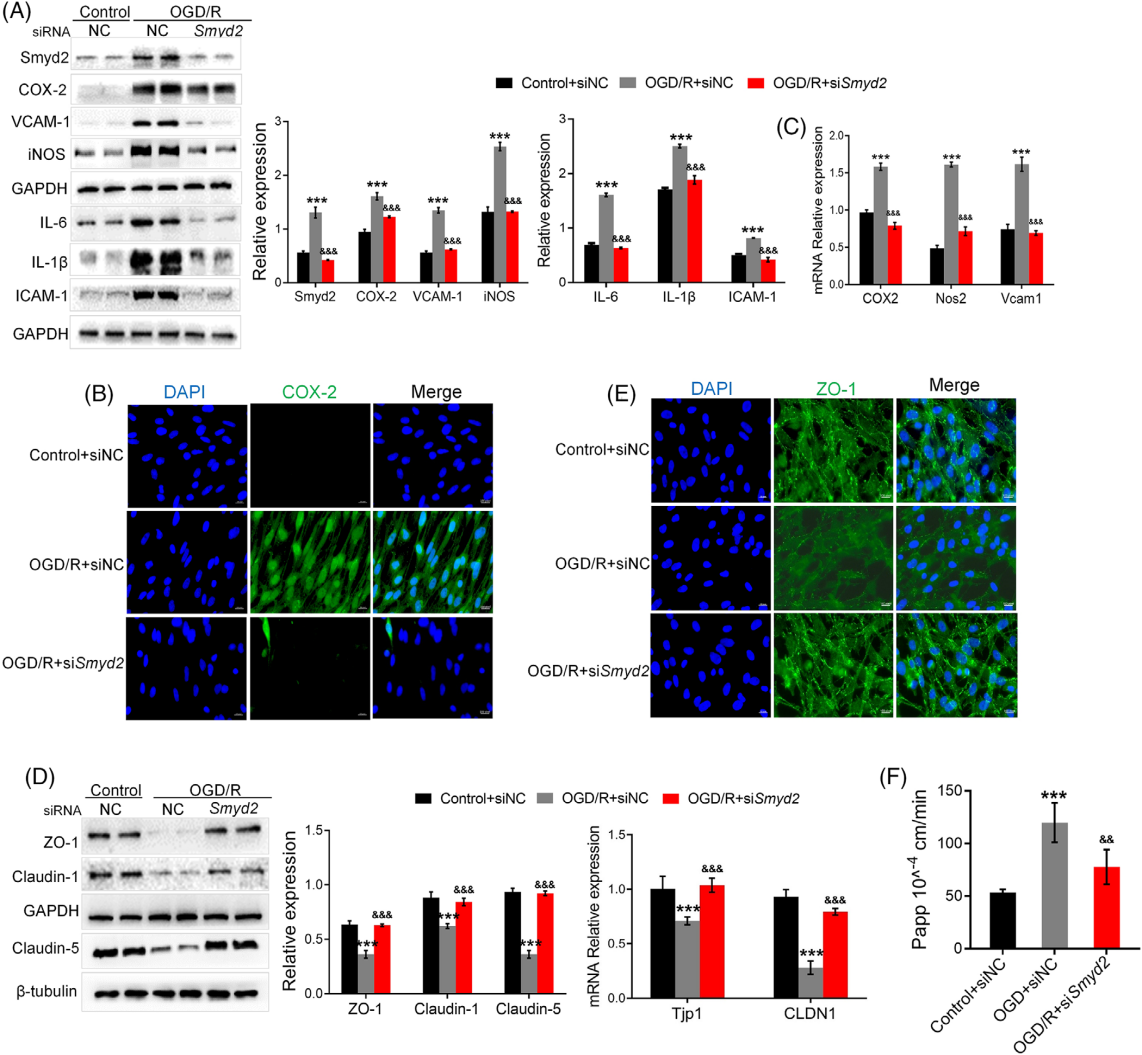
Loss of endothelial Smyd2 suppresses the inflammatory responses and endothelial cell barrier disruption after OGD/R. BMECs transfected with either control siRNA (siNC) or *Smyd2* siRNA (si*Smyd2*) were cultured for 48 h and subsequently treated with OGD for 4 h followed by 24 h of reoxygenation (OGD/R). Immunoblot analysis of Smyd2 and inflammation markers (iNOS, VCAM‐1, COX‐2, IL‐6, IL‐1β, ICAM‐1) (A); immunofluorescence staining of COX‐2, magnification ×200 (B); qRT‐PCR analysis of inflammation‐associated genes (*Nos2*, *Vcam1*, *COX2*) (C); immunoblot analysis and qRT‐PCR analysis of TJs‐associated proteins (claudin‐1, ZO‐1, claudin‐5) (D); immunofluorescence staining of ZO‐1, magnification ×200 (E); Na‐F permeability was detected (F). All data are presented as mean ± SD of four independent experiments; ^***^ *p* < .001, compared with the control+siNC group, ^&&^ *p* < .01, ^&&&^ *p* < .001, compared with the OGD/R+siNC group

To investigate the function of Smyd2 in OGD/R‐treated endothelial cells, we knocked down Smyd2 expression using siRNA. When Smyd2 was knocked down, the expression of inflammatory markers decreased (Figure 4A). In addition, immunofluorescence analyses revealed that the knockdown of Smyd2 reduced the expression of COX‐2 (Figure 4B). Consistent with these findings, knockdown of Smyd2 downregulated the mRNA levels of inflammation mediators (*Nos2*, *COX2* and *Vcam‐1*) (Figure 4C). Furthermore, knockdown of Smyd2 rescued the decrease in Claudin‐1/5 and ZO‐1 levels (Figure 4D). Similarly, immunostaining of ZO‐1 further showed that the knockdown of Smyd2 promoted the expression of ZO‐1 (Figure 4E). Loss of endothelial Smyd2 markedly reduced barrier leakage after OGD/R (Figure 4F). These results suggest that Smyd2 knockdown protects the endothelial cell barrier against OGD/R injury.

### 2.5 Sphk/S1PR is involved in Smyd2‐mediated endothelial cell barrier disruption in stroke

**FIGURE 5:**
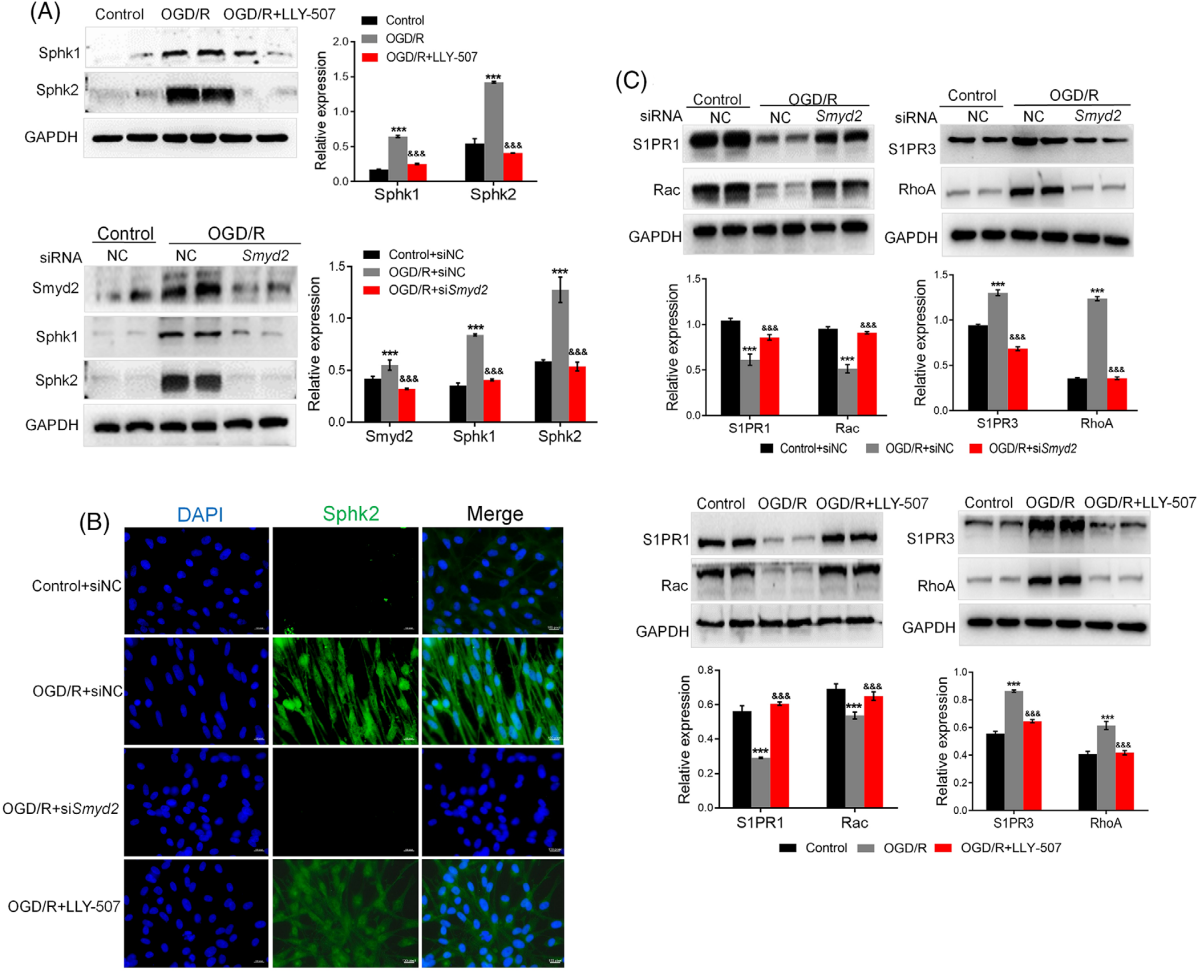
Sphk/S1PR is involved in Smyd2‐mediated endothelial cell barrier disruption in stroke. BMECs transfected with either control siRNA (siNC) or *Smyd2* siRNA (si*Smyd2*) or pre‐treated with LLY‐507, were subsequently subjected to oxygen‐glucose deprivation for 4 h and 24 h of reoxygenation (OGD/R). Immunoblot analysis of Sphk2, Sphk1, and Smyd2 (A); immunofluorescence staining of Sphk2, magnification ×200 (B); immunoblot analysis of S1PR3/RhoA and S1PR1/Rac (C). All data are presented as mean ± SD of four independent experiments; ^***^ *p* < .001, compared with the control group or the control+siNC group, ^&&&^ *p* < .001, compared with the OGD/R group or the OGD/R+siNC group

Given that S1PR1/3 plays an important role in the regulation of tight junctions between endothelial cells, we assessed whether Sphk/S1PR is involved in Smyd2‐mediated BMECs barrier disruption. The results showed that Sphk expression remarkably increased in OGD/R‐treated BMECs compared to the normal control. Interestingly, LLY‐507 or Smyd2 knockdown by siRNA reversed Sphk1/2 expression (Figure 5A). Further immunostaining showed that BMECs expressed Sphk2 with an increasing tendency, in response to OGD/R. In contrast, the blockade of Smyd2 downregulated the expression of Sphk2 (Figure 5B). S1PR1 has been reported to be responsible for enhanced barrier integrity, and reduced S1PR1 expression has been found to attenuate Rac1 activation and increase endothelial permeability. In sharp contrast, S1PR3/Rho induces endothelial permeability and inflammation. Consistent with this notion, we found that OGD/R not only downregulated S1PR1/Rac but also upregulated S1PR3/RhoA expression. However, the blockade of Smyd2 rescued protein expression of S1PR1/Rac and inhibited the upregulation of S1PR3/RhoA (Figure 5C). Consistent with these in vitro findings, Smyd2 knockdown decreased the expression of Sphk and S1PR3/RhoA and increased S1PR1/Rac expression in the brain of MCAO mice (Figure S2). In summary, Sphk/S1PR is involved in Smyd2‐mediated endothelial barrier disruption in stroke.

### 2.6 Inhibition of Sphk reduces the inflammatory response and endothelial barrier disruption after OGD/R

Because Sphk/S1PR is involved in Smyd2‐mediated endothelial barrier disruption, to further test the role of Sphk, we utilised Sphk1/2‐specific antagonists, PF‐543 and K145, to block S1P signalling during OGD/R‐treated BMECs. The results showed that both PF‐543 and K145 decreased the expression of inflammatory markers (Figure S3A). Simultaneously, PF‐543 and K145 upregulated ZO‐1 and Claudin‐1 (Figure S3B). In addition, pretreatment with PF‐543 or K145 inhibited the S1PR3/RhoA expression and restored the S1PR1/Rac expression (Figure S4A). Inhibition of Sphk also markedly reduced barrier leakage after OGD/R (Figure S4B). These results suggest that Sphk activity contributes to the inflammatory response and endothelial barrier disruption after OGD/R.

### 2.7 Overexpression of Smyd2 causes endothelial barrier disruption at least partly through the Sphk/S1PR pathway

**FIGURE 6:**
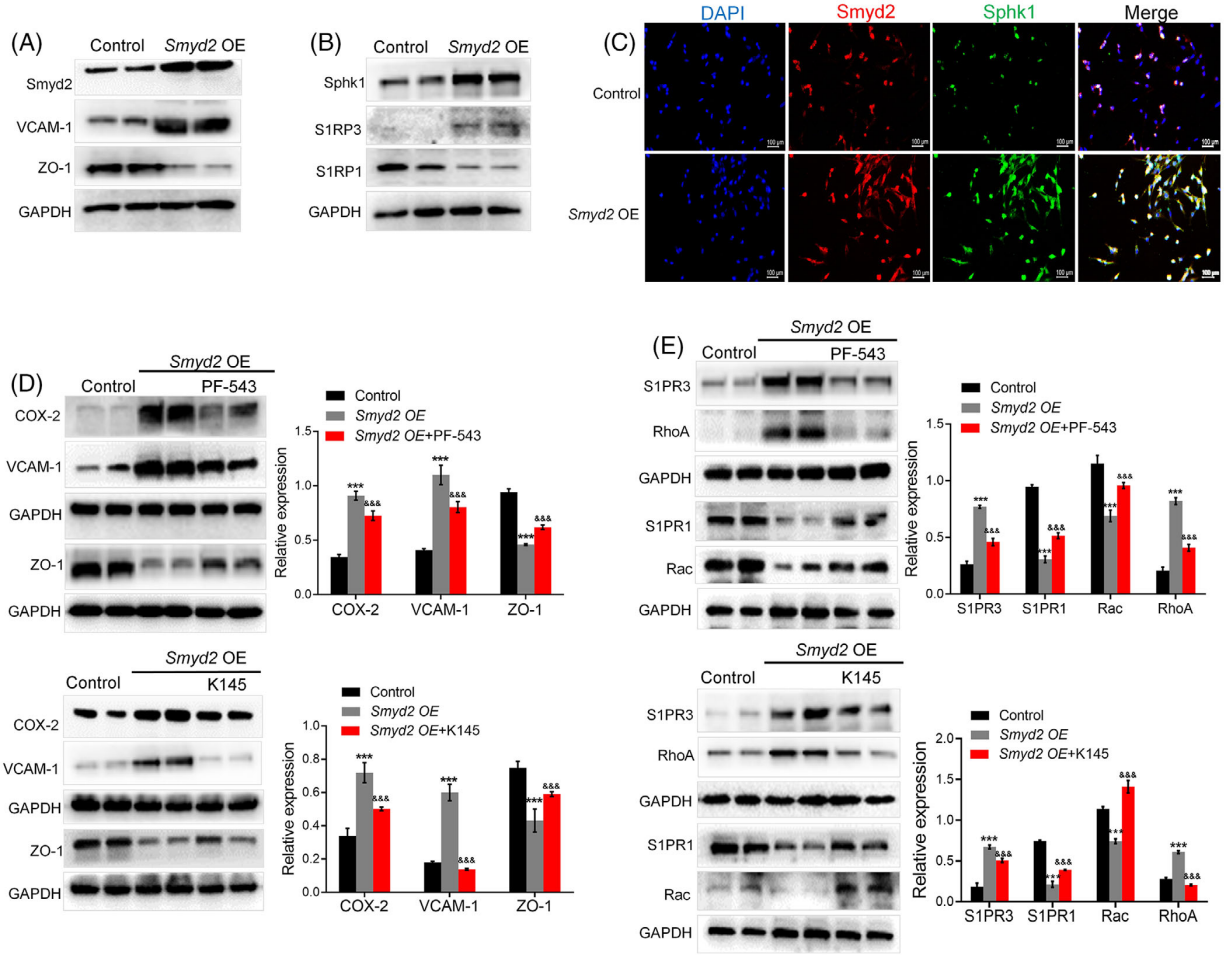
Overexpression Smyd2 causes endothelial cell barrier disruption through Sphk/S1PR. (A–C) Overexpression of Smyd2 causes endothelial cell inflammatory response and barrier disruption. BMECs were transfected with lentivirus‐mediated *Smyd2* cDNA (*Smyd2* OE) for 72 h, followed by an immunoblot analysis of VCAM‐1 and TJs‐associated protein ZO‐1 (A); Immunoblot analysis of Sphk1 and S1PR1/3 (B); immunofluorescence staining of Smyd2 and Sphk1 in BMECs. Scale bars: 100 μm (C). (D, E) Inhibition of Sphk reduces inflammatory response upon overexpression Smyd2. BMECs transfected with lentivirus‐mediated *Smyd2* cDNA (*Smyd2* OE) were cultured with PF‐543 (2 μmol/L) or K145 (10 μmol/L). Immunoblot analysis of inflammation markers (VCAM‐1, COX‐2) and TJs‐associated protein (ZO‐1) (D); Immunoblot analysis of S1PR3/RhoA and S1PR1/Rac (E). All data are presented as mean ± SD of four independent experiments; ^***^ *p* < .001, compared with the control group, ^&&&^ *p* < .001, compared with the *Smyd2* OE group

To determine whether Smyd2 is sufficient to disrupt the endothelial barrier, we selectively overexpressed Smyd2 in BMECs using lentivirus‐mediated transduction. The results showed that after Smyd2 overexpression, inflammatory responses and reduction in TJ protein levels were evident (Figure 6A). Unsurprisingly, overexpression of Smyd2 upregulated Sphk1 and S1PR3 but decreased S1PR1 expression (Figure 6B). In support of this idea, double‐immunofluorescence staining (Figure 6C) showed that overexpression of Smyd2 upregulated the expression of Sphk1. Next, we added an inhibitor of Sphk1 (PF‐543) and an inhibitor of Sphk2 (K145) to Smyd2‐overexpressing endothelial cells and found that both PF‐543 and K145 could reverse the upregulation of COX‐2 and VCAM‐1 induced by Smyd2 overexpression and rescue the ZO‐1 expression (Figure 6D). In addition, both PF‐543 and K145 not only eliminated the expression of S1PR3/RhoA, but also rescued the expression of S1PR1/Rac (Figure 6E). These results suggest that Smyd2 damages endothelial barrier integrity, at least partly through the Sphk/S1PR pathway.

### 2.8 Smyd2 regulates Sphk/S1PR through methylation‐mediated, ubiquitin‐dependent degradation

**FIGURE 7:**
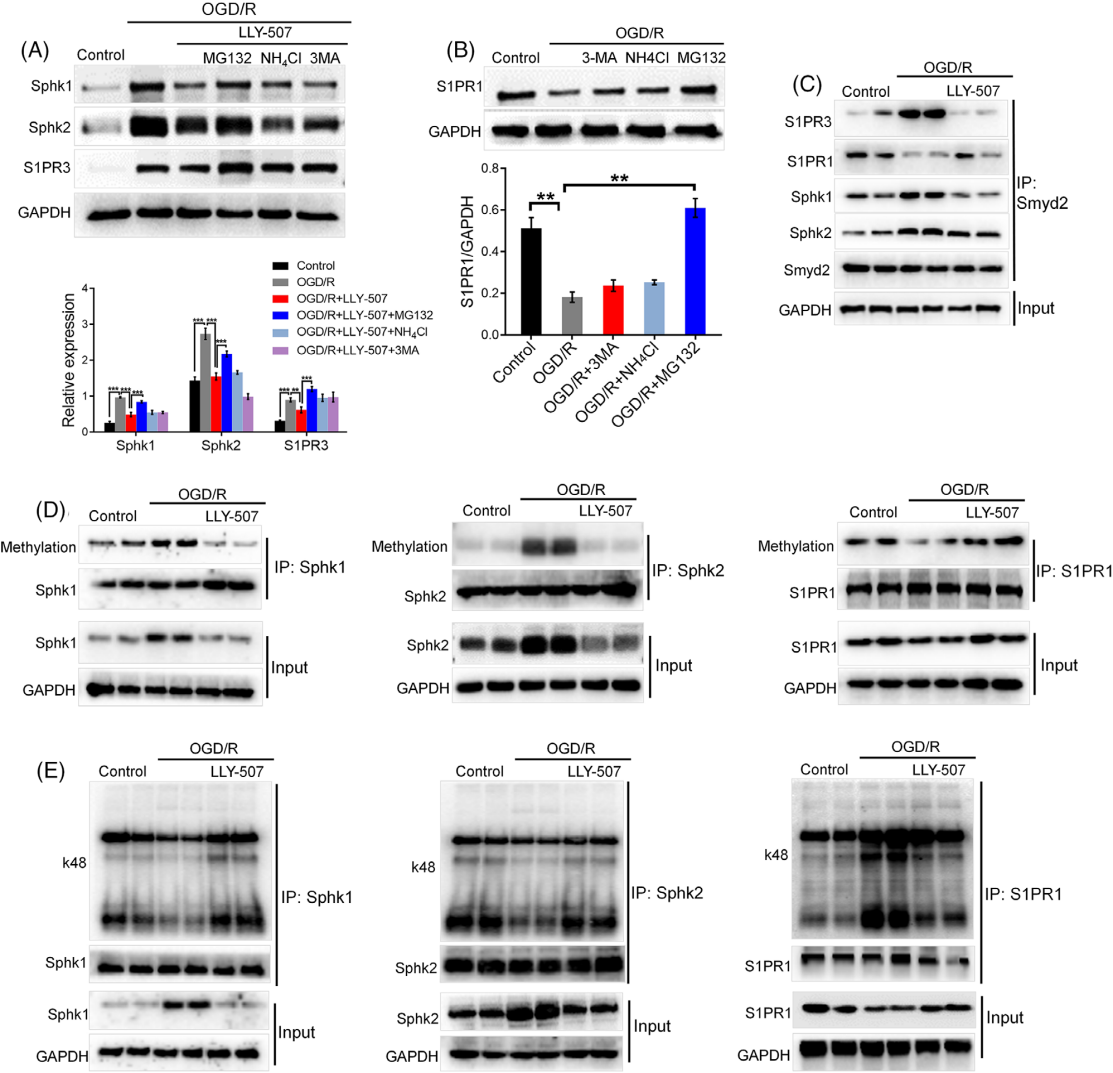
Smyd2 regulates Sphk/S1PR through methylation‐mediated, ubiquitin‐dependent degradation. (A, B) Smyd2 regulates Sphk/S1PR ubiquitin‐dependent degradation in OGD/R‐treated BMECs. BMECs were pre‐treated without or with MG132, NH_4_Cl, or 3‐MA together with LLY‐507 for 4 h and subsequently subjected to oxygen‐glucose deprivation 4 h followed by 24 h of reoxygenation (OGD/R) and an immunoblot analysis of Sphk1/2 and S1PR3 (A); BMECs were pre‐treated without or with MG132, NH_4_Cl, or 3‐MA for 4 h were subsequently subjected to OGD/R followed by immunoblot analysis of S1PR1 (B). (C–E) Smyd2 regulates K48 polyubiquitination of Sphk/S1PR through methylation modification. BMECs were pre‐treated with or without LLY‐507 for 4 h and subsequently subjected to OGD/R followed by co‐IP analysis of the interaction between Smyd2 and Sphk1/2, S1PR1/3 (C); Co‐IP analysis of Sphk1/2 and S1PR1 methylation levels (D); Co‐IP analysis of k48 polyubiquitination of Sphk1/2 and S1PR1 (E). All data are presented as mean ± SD of four independent experiments; ^**^ *p* < .01, ^***^ *p* < .001

To determine whether the changes in Sphk/S1PR expression were associated with k48‐linked polyubiquitination degradation in OGD/R‐induced BMECs, we first tested whether Smyd2 regulated Sphk/S1PR through a proteasome‐dependent degradation pathway. To this end, we found that MG132, a proteasome inhibitor, effectively rescued the decrease of Sphk and S1PR3 protein levels caused by LLY‐507 treatment in OGD/R‐induced BMECs (Figure 7A), on the contrary, the OGD/R‐decreased S1PR1 expression was restored by MG132 (Figure 7B). These findings suggest that proteasomal degradation is the most important degradation pathway for these targets.

To determine the regulatory effects of Smyd2 on Sphk/S1PR, we examined the interaction between Smyd2 and Sphk/S1PR. Our results showed that Smyd2 interacted with Sphk and S1PR3 in OGD/R‐induced BMECs, whereas the interaction between Smyd2 and S1PR1 was significantly decreased (Figure 7C). In addition, LLY‐507 efficiently decreased the interaction between Smyd2 and Sphk or S1PR3 but enhanced the interaction between Smyd2 and S1PR1 (Figure 7C). The finding that Smyd2 could methylate nonhistone proteins raised the question of whether the Sphk/S1PR pathway can be directly methylated by Smyd2. We analysed the status of Sphk/S1PR methylation in OGD/R‐treated cells. Figure 7D shows that OGD/R increased Sphk methylation and decreased S1PR1 methylation. More importantly, Smyd2 inhibition rescued the above‐mentioned changes in methylation levels. Taken together, these results indicate that Sphk/S1PR can be methylated by Smyd2.

Altogether, these findings suggest that Smyd2‐mediated methylation is responsible for Sphk/S1PR polyubiquitination and degradation. To test this hypothesis, we examined the ubiquitination levels of Sphk and S1PR1 in OGD/R‐induced BMECs. OGD/R treatment dramatically reduced the k48 polyubiquitination of Sphk, which was significantly restored by LLY‐507. Conversely, OGD treatment resulted in a markedly elevated k48 polyubiquitination level of S1PR1, which was significantly attenuated by LLY‐507 treatment (Figure 7E). Consistent with this finding, the methylation level was found to be responsible for the polyubiquitination and degradation of Sphk/S1PR. Taken together, these data clearly demonstrate that Smyd2‐mediated methylation targets Sphk/S1PR for polyubiquitination and proteasomal degradation.

**FIGURE 8:**
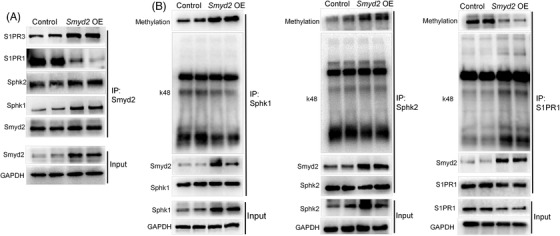
Overexpression Smyd2 regulates Sphk/S1PR methylation and K48 polyubiquitination. BMECs were transfected with lentivirus‐mediated *Smyd2* cDNA (*Smyd2* OE) for 72 h followed by a co‐IP analysis of the interaction between Smyd2 and Sphk1/2, S1PR1/3 (A); Co‐IP analysis of methylation and k48 polyubiquitination levels of Sphk1/2 and S1PR1 (B)

Next, to further confirm the mechanism between Smyd2 and Sphk‐S1PR1 signalling, we examined the Smyd2 interaction with S1PR1 in BMECs overexpressing Smyd2. We also identified an interaction between Smyd2 and Sphk or S1PR1/3 (Figure 8A). To test whether Smyd2 mediates Sphk/S1PR methylation in cells, Sphk/S1PR protein was immunoprecipitated, and methylation and k48 ubiquitination were analysed by western blotting. The results showed that overexpression of Smyd2 increased the level of Sphk methylation while decreasing the level of S1PR1 methylation (Figure 8B). Furthermore, overexpression of Smyd2 decreased k48 ubiquitination and protein degradation of Sphk but increased k48 ubiquitination and protein degradation of S1PR1 (Figure 8B). This finding suggests that overexpression of Smyd2 led to a substantial reduction of endogenous S1PR1 and upregulated Sphk1/2 proteins, in a methylase activity‐dependent manner in BMECs. These results show that Smyd2 regulates Sphk/S1PR through methylation‐mediated ubiquitin‐dependent degradation, which can promote endothelial barrier disruption after cerebral ischaemia.

## 3 DISCUSSION

During the reperfusion following ischaemic stroke, increased BBB permeability directly contributes to increased mortality.^24^ Therefore, restoration of the damaged BBB may be crucial in cerebral ischaemia. Our results demonstrate that endothelial cell‐derived Smyd2 mediates BBB disruption in response to ischaemia and can increase brain endothelial permeability by activating Sphk/S1PR signalling. To our knowledge, this study is the first to show the significance of endothelial‐derived Smyd2 in BBB breakdown and neurological disorders in MCAO mouse models.

The histone methyltransferase Smyd2 can affect a variety of biological processes and disease states by catalysing the transfer of methyl groups to histone or non‐histone protein substrates.^10^, ^25^ Thus, Smyd2 is involved in cancer via the methylation of key cancer proteins, including p53 and Rb. Here, we show that Smyd2 is elevated after MCAO, especially in the brain endothelium, implying that Smyd2 might play a significant role in brain pathology during reperfusion following ischaemic stroke. Consistently, using in vivo and in vitro ischaemia models, we demonstrated significant neuroprotective effects in the absence of Smyd2. Smyd2 pharmacological inhibitor improved the loss of endothelial integrity and relieved the inflammatory response in OGD‐induced BMECs. Furthermore, the functional recovery in Smyd2 knockdown mice was greater than that in WT mice, and the infarct area was also smaller in Smyd2 knockdown mice at 24 h post‐MCAO. Overexpression of Smyd2 caused an inflammatory response and increased endothelial permeability in BMECs. These data strongly support our hypothesis that Smyd2 derived from brain endothelial cells plays an important role in the regulation of ischaemic perfusion‐brain pathology, whereby the blockade of Smyd2 promotes functional recovery by maintaining BBB integrity.

Notably, recent studies have revealed that S1P signalling, a major bioactive lipid mediator, plays a potential role in BBB function and has attracted increasing research interest.^26^, ^27^ S1P is synthesised by Sphk1 and Sphk2 via sphingosine phosphorylation. We found that both Sphks were markedly upregulated in OGD/R‐treated BMECs and in the peri‐ischaemic cortex. This result is consistent with the idea that ischaemic conditions increase the production of S1P in several organs, such as the brain, heart and kidney.^28^ This result is also consistent with the idea that higher S1P concentrations can induce endothelial permeability. On the other hand, we showed that pharmacological Sphk1/2 inhibitors reduced the loss of endothelial integrity and inflammation response, which suggests that inhibition of excess S1P could provide a strategy to prevent BBB breakdown.

As recently reported, the physiological concentration of S1P promotes endothelial barrier integrity via S1PR1 and Rac1 activation, while higher S1P concentrations induce endothelial permeability and inflammation, via S1PR2 and the RhoA/ROCK pathway.^29^, ^30^ Similar to S1PR2, the activation of S1PR3 pathways promotes adherens junction assembly and stimulates stress fibres, resulting in a leaky barrier. Interestingly, in our study, the opposite trend was observed in S1PR1 and S1PR3 expression. Namely, S1PR1 was decreased and S1PR3 was increased in OGD/R‐treated BMECs and the peri‐ischaemic cortex. This result is consistent with the idea that endothelial cell S1PR1 supports BBB function and microvascular patency in the ischaemic brain,^31^ whereas S1PR3 could induce endothelial permeability and inflammation. Importantly, our findings demonstrate that stroke causes upregulation of Smyd2 in the brain. Increased Smyd2 impairs S1PR1 formation and induces S1PR3 production, which is detrimental to TJs and BBB dysfunction after stroke. Consistent with this, overexpression of Smyd2 in the endothelium promotes S1P production and increases endothelial cell permeability, resulting in BBB dysfunction. Furthermore, blocking Smyd2 restores endothelial integrity by suppressing the activation of the Sphk pathway and recovery of S1PR1, resulting in functional recovery. Taken together, these results show that Smyd2 enhances Sphk and S1PR3 expression and negatively regulates S1PR1 expression, resulting in endothelial dysfunction. These findings are the first to reveal that endothelial Smyd2 causes defects in endothelial cell TJs and BBB function by interrupting the S1P pathway.

Smyd2 has been shown to methylate histone H3K4 and H3K36, in addition to diverse non‐histone protein substrates,^11^, ^32^ and Smyd2‐methylated substrates have been found to mediate the function of Smyd2 in different diseases.^33^ Protein ubiquitylation and subsequent degradation by the proteasome drives protein degradation in cells and subsequently modulates a series of biological processes.^34^ First, we found that Sphk and S1PR1 degradation in BMECs was resistant to pretreatment with MG132, suggesting that ubiquitin‐dependent degradation is critical for Sphk and S1PR1 stability. Second, Smyd2 interacted with Sphk and S1PR1. Interestingly, Sphk methylation by Smyd2 stabilises the Sphk protein by inhibiting Sphk ubiquitylation and probably promotes Sphk activity. These results are consistent with the idea that Sphk can lead to higher concentrations of S1P signalling, thereby promoting BBB dysfunction after ischaemia. Furthermore, the inhibition of Smyd2 decreased Sphk methylation and promoted Sphk ubiquitylation, resulting in the downregulation of Sphk expression. In contrast, when Smyd2 was overexpressed or when BMECs were treated with OGD/R, methylated S1PR1 levels decreased. Importantly, methylation of S1PR1 is associated with distinct functions: decreased methylation of S1PR1 promotes k48‐ubiquitylation, resulting in S1PR1 degradation, whereas blocking Smyd2 recovers the methylation of S1PR1, inhibits S1PR1 k48‐ubiquitylation degradation, and promotes S1PR1 expression. These results are consistent with our observation that decreased S1PR1 levels disrupt tight junctions and increase permeability in BMECs upon OGD/R stimulation. Together, our findings demonstrate that Smyd2 regulates the Sphk/S1PR target gene in endothelial cell dysfunction, at least partly by affecting methylation status.

One of the limitations of this study is that it could not address the reasons why Smyd2 overexpression causes hypomethylation of S1PR1. Further studies are needed to investigate this mechanism and how it facilitates S1PR1 protein degradation upon OGD/R stimulation.

## 4 CONCLUSIONS

The present study provides previously unexplored evidence that endothelium‐derived Smyd2 mediates BBB disruption following stroke. Increased Smyd2 levels in the endothelium disrupt BBB integrity via the Sphk/S1PR signalling pathway, resulting in neuronal dysfunction. Given the substantial deleterious consequences of BBB dysfunction during stroke, Smyd2 represents a novel opportunity for the therapeutic improvement of cerebrovascular integrity.

## 5 MATERIALS AND METHODS

Detailed methods can be found in the Supplemental materials.

### 5.1 Animal studies

All animal experiments conformed to animal welfare guidelines and were approved by the Institutional Animal Care and Use Committee of Fudan University.

 The construction of Smyd2 heterozygous knockout (*Smyd2* ^+/–^) mice has been described previously.^35^ Male WT and *Smyd2* ^+/–^ mice that were 6–8 weeks old were subjected to middle cerebral artery occlusion (MCAO), except for those in the sham operation group. Mice were anesthetised with 1% pentobarbital sodium (50 mg/kg, i.p., Sigma) throughout the surgery. To induce transient ischaemia, suture occlusion was performed according to our previous method.^36^ Neurological deficits were evaluated 24 h after MCAO, with a neurological score in line with the criteria described by Bederson.^37^

### 5.2 Culture of rat BMECs and the in vitro OGD/R model

The BMECs were isolated as previously described.^38^ The beaded microvessel fragments and individual cells were plated onto gelatin‐coated dishes in DMEM/F12 medium supplemented with 20% FBS, 0.58 mg/ml L‐glutamin, 3 mg/ml glucose, penicillin and streptomycin. When the BMECs reached 90% confluence, endothelial cells were purified. Cells of 2–4 passages were used for experiments. When OGD/R was initiated, the culture was replaced with a glucose‐free medium and placed in < 0.1% oxygen in a hypoxia box. After 4 h, cells were cultured under normal conditions for 24 h. The Smyd2 inhibitor LLY‐507 (2 μM) or Sphk1/2 inhibitor (PF‐543, K145) was added 4 h before OGD and remained in the media during OGD/R.

### 5.3 Assessment of infarct volume

After neurological testing, the mice were sacrificed, after anaesthesia, and infarct volumes were assessed using *ImageJ* software, as previously described.^36^

### 5.4 Evans blue dye leakage assay

Evans blue (EB) perfusion was used to evaluate the BBB integrity. One hundred microliters of 2% Evans blue dye was injected through the tail vein of MCAO mice. After 2 h, the mice were administered transcardial perfusion with 50 ml of ice‐cold PBS, to wash out the intravascular dye. Then, the brains were cut into 2‐mm‐thick sections and photographed to observe the EB‐stained area. The concentration of EB was measured by spectrophotometry, and the results were expressed in μg/g of brain tissue.^39^

### 5.5 Brain water content assay

Immediately after sacrificing the mice, their brains were weighed, and then stored at 65°C for 72 h, dried and reweighed. The percentage water content was quantified as water content (%) = (wet weight ‐ dry weight)/wet weight ×100%.

### 5.6 Measurement of endothelial cell layer permeability

The paracellular permeability of sodium fluorescein (Na‐F) was measured to further assess the tight junction integrity of the endothelial cell layers.^40^ After treatment in each group, the NaF solution (10 mg/L) was added to the apical compartments, and 1 ml Hanks solution was added to the basolateral compartments. Then, the cells were pre‐incubated with 5% CO_2_ at 37°C for 60 min. After 1 h, transendothelial Na‐F transport was assessed. Quantification of Na‐F fluorescence was carried out using a Chameleon microplate reader (BioTek), and the permeability coefficient (Papp) was calculated.

### 5.7 Small interfering RNA transfection in vitro

*Smyd2* (5′‐GCUAUAUCGACCUGCUGUATT‐3′) and control (5′‐ UUCUCCGAACGUGUCACGUTT‐3′) small interfering RNA (siRNA) were obtained from GenePharma (China). Cells at ∼70% confluence were added to the prepared Lipofectamine RNA iMAX (Thermo Fisher Scientific) and siRNA *Smyd2* complexes. After incubating for 12 h, the medium was removed. Three days later, the Smyd2 knockdown efficiency was evaluated.

### 5.8 Overexpression lentivirus generation and infection

*Smyd2* cDNA sequences were obtained from the CCDS database and cloned into the lentiviral vector, pCDH‐CMV‐MCS‐EF1‐copGFP. cDNA expression vectors, recombinant plasmid and packaging vectors psPAX2 and PMD2.G were co‐transfected into HEK293 cells. Forty‐eight hours after transduction, the lentivirus‐containing medium was collected and centrifuged to remove cell debris with a 0.45 μm filter. The lentivirus was added to the culture medium of primary BMECs with polybrene. After 24 h, the medium was replaced with fresh DMEM, and the cells were incubated for an additional 48 h.

### 5.9 Co‐immunoprecipitation

Different treated endothelial cells were lysed in a buffer composed of 20 mmol/L Tris‐HCl (pH 8), 1% Nonidet P‐40 (NP‐40), 10% glycerol, 137 mmol/L NaCl, 2 mmol/L EDTA and a protease inhibitor cocktail. Two micrograms of antibody and protein A/G agarose beads were added to the cell lysate, and the lysates were incubated overnight at 4°C. The beads were washed 3–5 times, and the proteins were separated by SDS‐PAGE.

### 5.10 Statistical analysis

Data are expressed as mean ± SD. Comparisons among groups were analysed by one‐way ANOVA with the Tukey‐Kramer post hoc test, and comparisons between two groups were performed using the unpaired Student's *t*‐test. All analyses were performed using GraphPad Prism 8.0, and statistical significance was defined at a *p* value of < .05.

## CONFLICT OF INTEREST

The authors of this manuscript have no conflicts of interest to disclose.

## Supporting information

Supporting information
