# Supplementary material for: Histone methyltransferase Smyd2 contributes to blood‐brain barrier breakdown in stroke
Source: Clin Transl Med. 2022 Mar 16;12(3):e761. doi: 10.1002/ctm2.761 (PMC8926904; doi:10.1002/ctm2.761)
Supplement: Supplementary file 1 — Supporting information [file CTM2-12-e761-s001.docx]

**Supplementary materials and methods**

**Materials**

MG132, NH_4_Cl, 3-MA, 2,3,5-triphenyltetrazolium chloride (TTC) were obtained from Sigma (Sigma-Aldrich, USA). LLY-507 (Smyd2 inhibitor) was obtained from BioChemPartner (China). PF-543 (Sphk1 inhibitor) and K145 (Sphk2 inhibitor) were purchased from MCE (MCE, USA).

**Immunohistochemical staining**

Tissues or cell slices were treated with autoclaving at 121°C for 15 min in 0.01 mol/L citrate-buffered saline (pH 6.0) for antigen retrieval, and were then immersed in 3% H_2_O_2_ for 30 min at room temperature to block the endogenous peroxidase activity. After deactivation, 10% normal goat serum was used to block nonspecific binding of the immunological reagents. After incubation of the antibodies against CD45 at 4°C overnight, each slide was rinsed three times in PBS and incubated with biotinylated anti-rabbit IgG and HRP-streptavidin at room temperature according to the immunohistochemical staining kit (ThermoFisher Scientific), stained with DAB substrate. Finally, nuclear counterstaining was done using hematoxylin.

**Immunofluorescence staining**

After the stimulation as indicated, cells were fixed with pre-warmed 4% paraformaldehyde and then permeabilized for 10 min using 1% Triton X-100 in dissolved in PBS followed by incubation with the primary antibodies at 4°C overnight. The cells were washed three times with PBS and incubated with Fluor-conjugated secondary antibody (ThermoFisher Scientific). The nucleus was stained with 4',6-diamidino-2-phenylindole (DAPI) to locate the cell. Fluorescent pictures were obtained with a Zeiss fluorescence microscope.

**Western blot analysis**

Tissue samples from the left ischemic hemisphere or culture cells were pooled and homogenized with a homogenizer or by sonication in ristocetin-induced platelet aggregation (RIPA) buffer containing protease and phosphatase inhibitors (Complete Protease Inhibitor Cocktail and PhosSTOP Phosphatase Inhibitor Cocktail), and then centrifuged at 12,000 g for 10 min at 4°C. The supernatant was collected. The total protein concentration in each sample was determined with a bicinchoninic acid (BCA) assay according to the manufacturer’s instructions. Equal amount of protein samples was separated by SDS-polyacrylamide gel electrophoresis and transferred to nitrocellulose membranes (Millipore). The membranes were blocked with 5% skimmed milk (wt/vol) in Tris-buffered saline supplemented with 0.1% Tween 20 (TBST) for 1 hr at room temperature and then incubated with the corresponding primary antibodies: rabbit anti-VCAM-1 (1:1000; Proteintech); rabbit anti-COX-2 (1:1000; Proteintech); rabbit anti-iNOS (1:1000; Cell Signal Technology); rabbit anti-Zona Occludens-1 (ZO-1, 1:1000; Proteintech); rabbit anti-Claudin-1 (1:1000; Proteintech); rabbit anti-Bax (1:500; Cell Signaling Technology); rabbit anti-Bcl-2 (1:500; Cell Signaling Technology); rabbit anti-Caspase 3 (1:1000; Cell Signaling Technology); mouse anti-Caspase 9 (1:1000; Proteintech); mouse anti-p53 (1:1000; Proteintech); mouse anti-ICAM-1 (1:500; Santa); rabbit anti-IL-6 (1:1000; Proteintech); rabbit anti-IL-1β (1:1000; Proteintech); rabbit anti-Sphk1 (1:1000; Proteintech); rabbit anti-Sphk2 (1:1000; Proteintech); rabbit anti-S1PR1 (1:1000; Proteintech); rabbit anti-S1PR3 (1:1000; Proteintech); rabbit anti-Rac (1:500; Proteintech); rabbit anti-RhoA (1:1000; Proteintech); rabbit anti-Smyd2 (1:500; Cell Signaling Technology); rabbit anti-methylation (1:1000; Cell Signaling Technology); rabbit anti-k48 (1:1000; Cell Signaling Technology); and mouse anti-GAPDH (1:20000; Proteintech) at 4°C overnight. The members were then washed and incubated for 1 hr at room temperature with the speciesappropriate horseradish peroxidase (HRP)-labeled secondary antibody (1:5000; Jackson ImmunoResearch Inc., USA). Protein-specific signals were detected using a Bio-Rad Imager (Bio-Rad, Hercules, CA, USA), and the bands were quantified by densitometric analysis (ImageJ software, NIH). The relative amounts of proteins were normalized against GAPDH.

**RNA extraction and quantitative real-time polymerase chain reaction (qRT-PCR)**

Total RNA was isolated from the cells or tissues using TRIzol reagent (Takara Bio Inc., China). Reverse transcription was performed according to the instruction of the Prime RT Master Mix kit (Takara Bio Inc., China). Quantitative real-time PCR was performed in triplicate using SYBR Premix EX Taq II (Yeasen Biotech Co., Ltd. China). The sequences of primers were shown in **Table S1**.


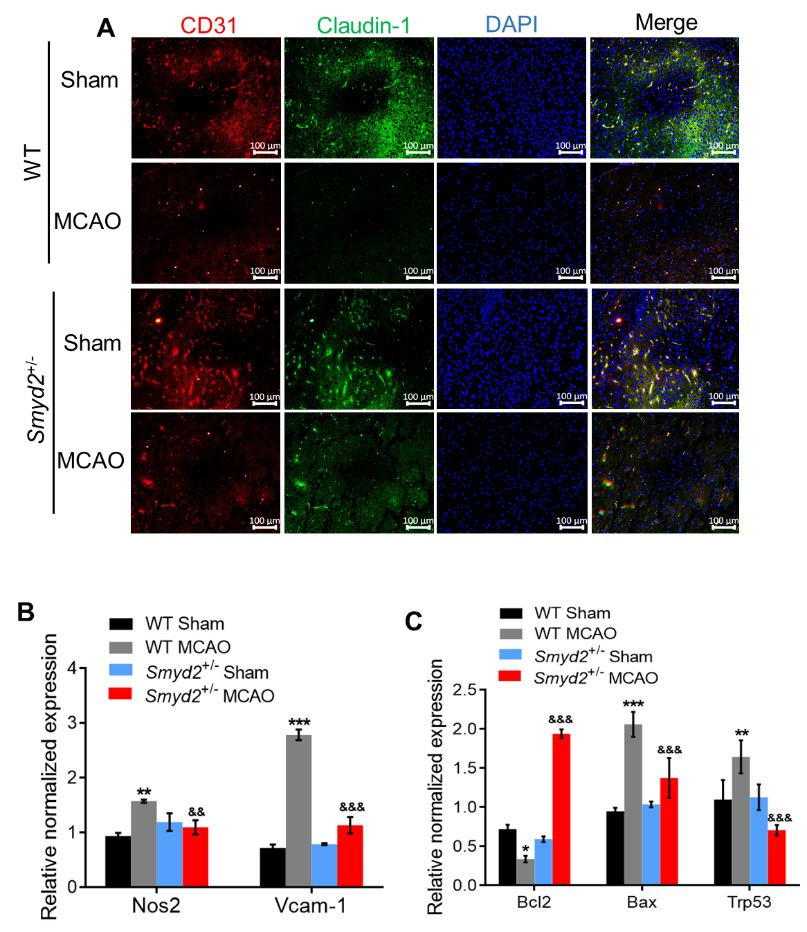


**Fig. S1 Knockdown Smyd2 increases** Claudin-1 expression, **reduces apoptosis and inflammation genes expression.** WT and *Smyd2*^+/-^ mice were subjected to 90 min of MCAO and 24 h of reperfusion. Immunofluorescence staining of CD31 and Claudin-1 in brain of mice after MCAO. Scale bars: 100 μm (**A**); qRT-PCR analysis of inflammation-associated genes (*Nos2*, *Vcam1*) in brain of mice after MCAO (**B**); qRT-PCR analysis of apoptosis-associated genes (*Bax*, *Bcl2*, *Trp53*) in brain of mice after MCAO (**C**). All data are presented as mean ± S.D, n=6/group, ^*^*p* < 0.05, ^**^*p* < 0.01, ^***^*p* < 0.001 compared with WT-Sham group, ^&&^*p* < 0.01, ^&&&^*p* < 0.001 compared with WT-MCAO group.


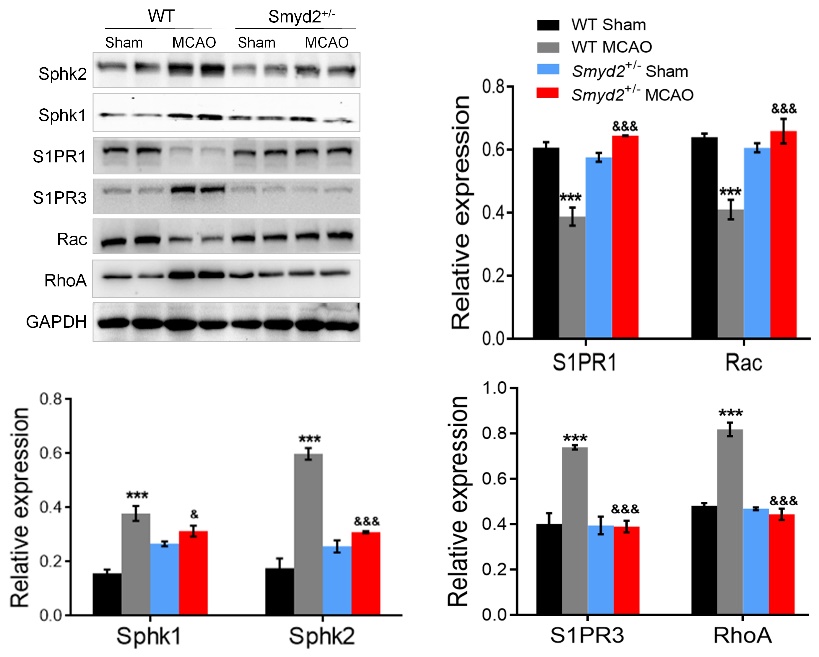


**Fig. S2 knockdown Smyd2 regulates Sphk/S1PR signaling.** WT and *Smyd2*^+/-^ mice were subjected to 90 min of MCAO and 24 hr of reperfusion, immunoblot analysis of the protein expressions of Sphk1/2, S1PR1/3, Rac and RhoA. Data are presented as mean ± S.D, n=6/group; ^***^*p* < 0.001 compared with WT-Sham group, ^&^*p* < 0.05, ^&&&^*p* < 0.001 compared with WT-MCAO group.

**
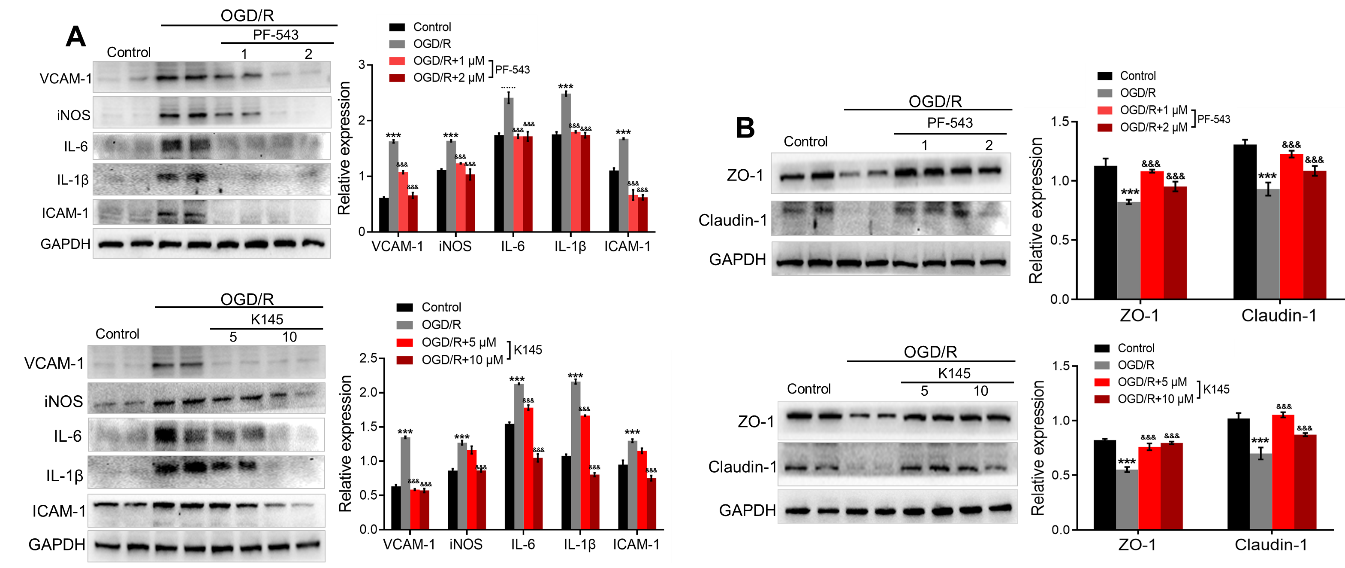
**

**Fig. S3 Inhibition of Sphk1 or Sphk2 reduces inflammation response, and promotes tight junctions (TJs) in BMECs.** BMECs pre-treated with PF145 or K145 for 4 hr were subjected to oxygen-glucose deprivation 4 hr and 24 hr of reoxygenation (OGD/R), immunoblot analysis of inflammation markers (iNOS, VCAM-1, ICAM-1, IL-1β, IL-6) (**A**); Immunoblot analysis of TJs-associated proteins (ZO-1, Claudin-1) (**B**). All data are presented as mean ± S.D of four independent experiments; ^***^*p* < 0.001 compared with Control, ^&&&^*p* < 0.001 compared with OGD/R group.


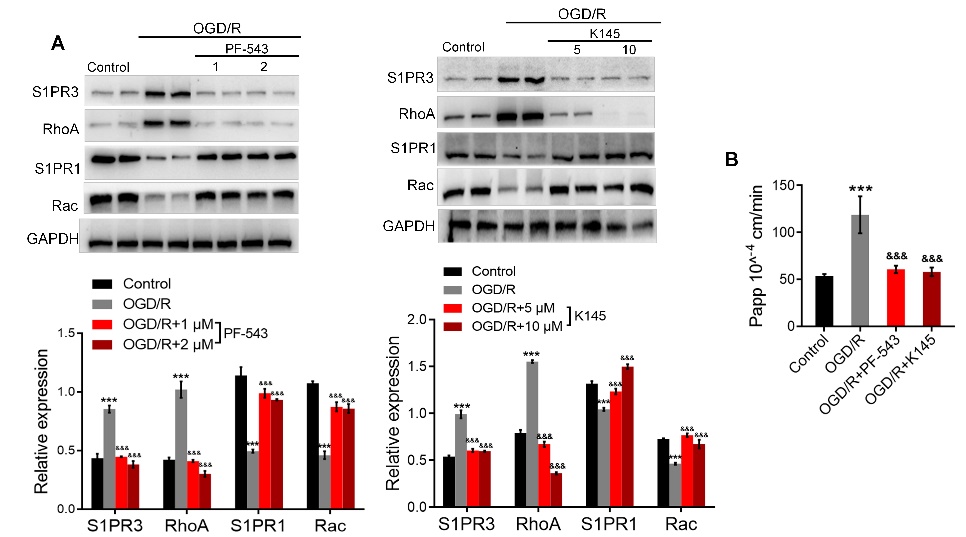


**Fig. S4 Inhibition of Sphk regulates endothelial barrier.** BMECs pre-treated with PF145 or K145 for 4 hr were subjected to oxygen-glucose deprivation 4 hr and 24 hr of reoxygenation (OGD/R), immunoblot analysis of the protein expressions of S1PR1/3, Rac and RhoA (**A**); Na-F permeability was analyzed (**B**). All data are presented as mean ± S.D of four independent experiments; ^***^*p* < 0.001 compared with Control group, ^&&&^*p* < 0.001 compared with OGD/R group.

**Table S1** Primers used for qRT-PCR validation.

| Gene name | Primer name | Primer sequence (5´ to 3´) |
| --- | --- | --- |
| Nos2 | Mouse _ Nos2_F | TTCACAGCTCATCCGGTACG |
|  | Mouse _ Nos2_R | GCCTAGGTCGATGCACAACT |
| Vcam1 | Mouse _ Vcam-1_F | GCTGCGAGTCACCATTGTTC |
|  | Mouse _ Vcam-1_R | ACTTCGTTCCAGCTTCCCAG |
| Trp53 | Mouse _p53_F | AGACAGGCAGACTTTTCGCC |
|  | Mouse _p53_R | TCTTCTTCTGTACGGCGGTC |
| Bax | Mouse _ Bax _F | GCAGGGAGGATGTCTGGGGAGA |
|  | Mouse _ Bax _R | TCCAGACAAGCAGCCGCTCACG |
| Bcl2 | Mouse _ Bcl-2_F | CAGGCTGGAAGGAGAAGAT |
|  | Mouse _ Bcl-2_R | CGGGAGAACAGGGTATGA |
| GAPDH | Mouse _GAPDH_ F | CTTCTCTTGTGACAAAGTGGACAT |
|  | Mouse _GAPDH_ R | TTCTCAGCCTTGACTGTGCC |
| Tjp1 | Mouse _ ZO-1_ F | CTCTTGCTGGCCCTAAACCT |
|  | Mouse _ ZO-1_ R | TTCGGGTGGCTTCACTTGAG |
| CLDN1 | Mouse _ Claudin-1_ F | TATGACCCCTTGACCCCCAT |
|  | Mouse _ Claudin-1_ R | AGAGGTTGTTTTCCGGGGAC |
| Tjp1 | Rat _ ZO-1_ F | AGAAACCTCAAGCGAAGCCA |
|  | Rat _ ZO-1_ R | TCAGTTTCGGGTTTCCCCTT |
| CLDN1 | Rat _ Claudin-1_ F | TGGGGACAACATCGTGACTG |
|  | Rat _Claudin-1_ R | CCCCAGCAGGATGCCAATTA |
| Nos2 | Rat _ Nos2_F | CAGCCTGTGAGACGTTCGAT |
|  | Rat _ Nos2_R | CCCATGTTGCGTTGGAAGTG |
| Vcam1 | Rat _ Vcam-1_F | CTGCACGGTCCCTAATGTGT |
|  | Rat _ Vcam-1_R | CAAGAGCTTTCCCGGTGTCT |
| Cox2 | Rat _ COX-2_F | TGCATTCTTTGCCCAGCACT |
|  | Rat _ COX-2_R | AAAGGCGCAGTTTACGCTGT |
| GAPDH | Rat _GAPDH_ F | TCAACGGCACAGTCAAGG |
|  | Rat _GAPDH_ R | AGCATCAAAGGTGGAAGAAT |
